# Estimation of alternative splicing isoform frequencies from RNA-Seq data

**DOI:** 10.1186/1748-7188-6-9

**Published:** 2011-04-19

**Authors:** Marius Nicolae, Serghei Mangul, Ion I Măndoiu, Alex Zelikovsky

**Affiliations:** 1 Department of Computer Science & Engineering, University of Connecticut,371 Fairfield Rd., Unit 2155, Storrs, CT 06269-2155, USA; 2 Computer Science Department, Georgia State University, University Plaza, Atlanta, Georgia 30303, USA

## Abstract

**Background:**

Massively parallel whole transcriptome sequencing, commonly referred as RNA-Seq, is quickly becoming the technology of choice for gene expression profiling. However, due to the short read length delivered by current sequencing technologies, estimation of expression levels for alternative splicing gene isoforms remains challenging.

**Results:**

In this paper we present a novel expectation-maximization algorithm for inference of isoform- and gene-specific expression levels from RNA-Seq data. Our algorithm, referred to as IsoEM, is based on disambiguating information provided by the distribution of insert sizes generated during sequencing library preparation, and takes advantage of base quality scores, strand and read pairing information when available. The open source Java implementation of IsoEM is freely available at http://dna.engr.uconn.edu/software/IsoEM/.

**Conclusions:**

Empirical experiments on both synthetic and real RNA-Seq datasets show that IsoEM has scalable running time and outperforms existing methods of isoform and gene expression level estimation. Simulation experiments confirm previous findings that, for a fixed sequencing cost, using reads longer than 25-36 bases does not necessarily lead to better accuracy for estimating expression levels of annotated isoforms and genes.

## Background

Ubiquitous regulatory mechanisms such as the use of alternative transcription start and polyadenylation sites, alternative splicing, and RNA editing result in multiple messenger RNA (mRNA) isoforms being generated from a single genomic locus. Most prevalently, alternative splicing is estimated to take place for over 90% of the multi-exon human genes across diverse cell types [1], with as much as 68% of multi-exon genes expressing multiple isoforms in a clonal cell line of colorectal cancer origin [2]. Not surprisingly, the ability to reconstruct full length isoform sequences and accurately estimate their expression levels is widely believed to be critical for unraveling gene functions and transcription regulation mechanisms [3].

Three key interrelated computational problems arise in the context of transcriptome analysis: *gene expression level estimation (GE), isoform expression level estimation (IE)*, and *novel isoform discovery (ID)*. Targeted GE using methods such as quantitative PCR has long been a staple of genetic studies. The completion of the human genome has been a key enabler for genome-wide GE performed using expression microarrays. Since expression microarrays have limited capability of detecting alternative splicing events, specialized splicing arrays have been developed for genome-wide interrogation of both annotated exons and exon-exon junctions. However, despite sophisticated deconvolution algorithms [4,5], the fragmentary information provided by splicing arrays is typically insufficient for unambiguous identification of full-length transcripts [6,7]. Massively parallel whole transcriptome sequencing, commonly referred to as RNA-Seq, is quickly replacing microarrays as the technology of choice for performing GE due to their wider dynamic range and digital quantitation capabilities [8]. Unfortunately, most RNA-Seq studies to date still ignore alternative splicing or, similar to splicing array studies, restrict themselves to surveying the expression levels of exons and exon-exon junctions. The main difficulty in inferring expression levels for full-length isoforms lies in the fact that current sequencing technologies generate short reads (from few tens to hundreds of bases), many of which cannot be unambiguously assigned to individual isoforms.

### Related work

RNA-Seq analyses typically start by mapping sequencing reads onto the reference genome, transcript libraries, exon-exon junction libraries, or combinations thereof. Early RNA-Seq studies have recognized that limited read lengths result in a significant percentage of so called *multireads*, i.e., reads that map equally well at multiple locations in the genome. A simple (and still commonly used) approach is to discard multireads, and estimate expression levels using only the so called *unique *reads. Mortazavi et al. [9] proposed a multiread "rescue" method whereby initial gene expression levels are estimated from unique reads and used to fractionally allocate multireads, with final expression levels obtained by re-estimation based on total counts obtained after multiread allocation. An expectation-maximization (EM) algorithm that extends this scheme by repeatedly alternating between fractional read allocation and re-estimation of gene expression levels was recently proposed in [10].

A number of recent works have addressed the IE problem, namely isoform expression level estimation from RNA-Seq reads. Under a simplified "exact information" model, [7] showed that neither single nor paired read RNA-Seq data can theoretically guarantee unambiguous inference of isoform expression levels, although paired reads may be sufficient to deconvolute expression levels for the majority of annotated isoforms. The key challenge in IE is accurate assignment of ambiguous reads to isoforms. Compared to the GE context, read ambiguity is much more significant, since it affects not only multireads, but also reads that map at a unique genome location expressed in multiple isoforms. Estimating isoform expression levels based solely on unambiguous reads, as suggested, e.g., in [2], results in splicing-dependent biases similar to the transcript-length bias noted in [11], further complicating the design of unbiased differential expression tests based on RNA-Seq data. To overcome this difficulty, [12] proposed a Poisson model of single-read RNA-Seq data explicitly modeling isoform frequencies. Under their model, maximum likelihood estimates are obtained by solving a convex optimization problem, and uncertainty of estimates is obtained by importance sampling from the posterior distribution. Li et al. [13] introduced an expectation-maximization (EM) algorithm similar to that of [10] but applied to isoforms instead of genes. Unlike the method of [12], which estimates isoform frequencies only from reads that map to a unique location in the genome, the algorithm of [13] incorporates multireads as well. The IE problem for single reads is also tackled in [14], who propose an EM algorithm for inferring isoform expression levels from the read coverage of exons (reads spanning exon junctions are ignored).

The related novel isoform discovery (ID) problem is also receiving much interest in the literature. Although showing encouraging results, *de novo *transcriptome assembly algorithms such as [15-17] have difficulties in identifying transcripts with moderate coverage. Very recently, [18-20] proposed genome-assisted (i.e., mapping based) methods for simultaneously solving ID and IE based on paired RNA-Seq reads. The method of Feng et al. [18] generates isoform candidates from the splicing graph derived from annotations and reads spanning exon-exon junctions. After discarding multireads, [18] formulates IE for a given set of isoforms as a convex quadratic program (QP) that can be efficiently solved for each gene locus. The set of isoform candidates is iteratively refined until the *p*-value of the objective value of the QP, assumed to follow a *χ*^2 ^distribution, exceeds an empirically selected threshold of 5%. Pair read information is not directly used in isoform frequency estimation, contributing only as secondary data to filter out false positives in the process of isoform selection. As in [18], Guttman et al. [19] construct a splicing graph from the mapped reads and filter candidate isoforms using paired-end information. Isoform specific expression levels are inferred using the method of [9]. After performing spliced alignment of (paired) reads onto the genome using TopHat [21], the method of Trapnell et al. [20], referred to as Cufflinks, constructs a read overlap graph and generates candidate isoforms by finding a minimal size path cover via a reduction to maximum matching in a weighted bipartite graph. Reads that match equally well multiple locations in the genome are fractionally allocated to these locations, and estimation is then performed independently at different transcriptional loci, using an extension to paired reads of the methods in [12].

### Our contributions

In this paper we focus on the IE problem, namely estimating isoform expression levels (interchangeably referred to as frequencies) from RNA-Seq reads, under the assumption that a complete list of candidate isoforms is available. Projects such as [22] and [23] have already assembled large libraries of full-length cDNA sequences for humans and other model organisms, and the coverage of these libraries is expected to continue to increase rapidly following ultra-deep paired-end transcriptome sequencing projects such as [19,20] and the widely anticipated deployment of third-generation sequencing technologies such as [24,25], which deliver reads with significantly increased length. Inferring expression at isoform level provides information for finer-resolution biological studies, and also leads to more accurate estimates of expression at the gene level by allowing rigorous length normalization. Indeed, as shown in the 'Experimental results' section, genome-wide gene expression level estimates derived from isoform level estimates are significantly more accurate than those obtained directly from RNA-Seq data using isoform-oblivious GE methods such as the widely used counting of unique reads, the rescue method of [9], or the EM algorithm of [10].

Our main contribution is a novel expectation-maximization algorithm for isoform frequency estimation from any mixture of single and paired RNA-Seq reads. A key feature of our algorithm, referred to as IsoEM, is that it exploits information provided by the distribution of insert sizes, which is tightly controlled during sequencing library preparation under current RNA-Seq protocols. Such information is not modeled in the "exact" information models of [6,7], challenging the validity of their negative results. Guttman et al. [19] take into account insert lengths derived from paired read data, but only for filtering candidate isoforms in ID. Trapnell et al. [20] is the only other work we are aware of that exploits this information for IE, in conjunction with paired read data. We show that modeling insert sizes is highly benefficial for IE even for RNA-Seq data consisting of single reads. Insert sizes contribute to increased estimation accuracy in two different ways. On one hand, they can help disambiguating the isoform of origin for the reads. In IsoEM, insert lengths are combined with base quality scores, and, if available, read pairing and strand information to probabilistically allocate reads to isoforms during the expectation step of the algorithm. As in [13], the genomic locations of multireads are also resolved probabilistically in this step, further contributing to improved overall accuracy compared to methods that ignore or fractionally pre-allocate multireads. On the other hand, insert size distribution is used to accurately adjust isoform lengths during frequency re-estimation in the maximization step of the IsoEM algorithm.

We also present the results of comprehensive experiments conducted to assess the performance of IsoEM on both synthetic and real RNA-Seq datasets. These results show that IsoEM consistently outperforms existing methods under a wide range of sequencing parameters and distribution assumptions. We also report results of experiments empirically evaluating the effect of sequencing parameters such as read length, read pairing, and strand information on estimation accuracy. Our experiments confirm the surprising finding of [13] that, for a fixed total number of sequenced bases, longer reads do not necessarily lead to better accuracy for estimation of isoform and gene expression levels.

## Methods

### Read mapping

As with many RNA-Seq analyses, the first step of IsoEM is to map the reads. Our approach is to map them onto the library of known isoforms using any one of the many available ungapped aligners (we used Bowtie [26] with default parameters in our experiments). An alternative strategy is to map the reads onto the genome using a spliced alignment tool such as TopHat [21], as done, e.g., in [19,20]. However, preliminary experiments with TopHat resulted in fewer mapped reads and significantly increased mapping uncertainty, despite providing TopHat with a complete set of annotated junctions. Since further increases in read length coupled with improvements in spliced alignment algorithms could make mapping onto the genome more attractive in the future, we made our IsoEM implementation compatible with both mapping approaches by always converting read alignments to genome coordinates and performing all IsoEM read-isoform compatibility calculations in genome space.

### Finding read-isoform compatibilities

**Figure 1:**
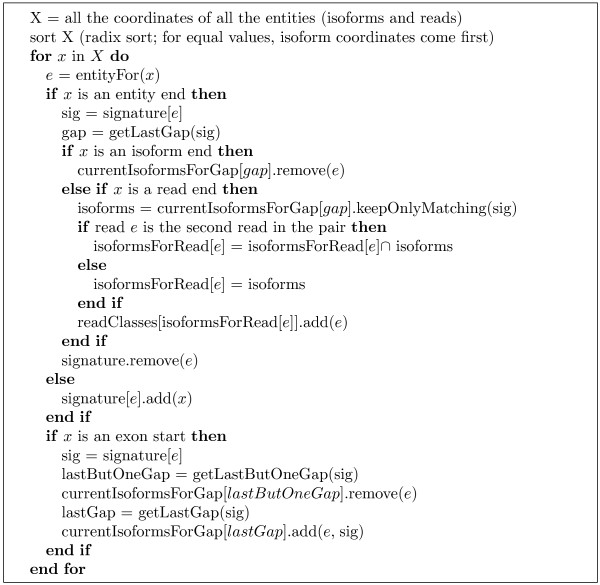
**The algorithm for identifying isoforms compatible with reads**.

The candidate set of isoforms for each read is obtained by combining all genome coordinates of reads and isoforms, sorting them and using a line sweep technique to detect read-isoform compatibilities (see Figure 1). As detailed below, during the line sweep reads are grouped into equivalence classes defined by their isoform compatibility sets; this speeds up the E-step of the IsoEM algorithm by allowing the processing of an entire read class at once.

Some of the reads match multiple positions in the genome, which we refer to as *alignments *(for paired end reads, an alignment consists of the positions where the two reads in the pair align with the genome). Each alignment *a *can in turn be compatible with multiple isoforms that overlap at that position of the genome. During the line sweep, we compute the relative "weight" of assigning a given read/pair *r *to isoform *j *as *w_r, j _*= ∑*_a _Q_a_F_a_O_a_*, where the sum is over all alignments of *r *compatible with *j*, and the factors of the summed products are defined as follows:

• *Q_a _*represents the probability of observing the read from the genome locations described by the alignment. This is computed from the base quality scores as , where  if position *k *of alignment *a *matches the reference genome sequence and 0 otherwise, while *ε_k _*denotes the error probability of *k*-th base of *r*.

• For paired end reads, *F_a _*represents the probability of the fragment length needed to produce alignment *a *from isoform *j*; note that the length of this fragment can be inferred from the genome coordinates of the two aligned reads and the available isoform annotation. For single reads, we can only estimate an upperbound *u *on the fragment length: if the alignment is on the same strand as the isoform then *u *is the number of isoform annotated bases between the 5*' *end of the aligned read and the 3*' *end of the isoform, otherwise *u *is the number of isoform annotated bases between the 5*' *end of the aligned read and the 5*' *end of the isoform. In this case *F_a _*is defined as the probability of observing a fragment with length of *u *bases or fewer.

• *O_a _*is 1 if alignment *a *of *r *is consistent with the orientation of isoform *j*, and 0 otherwise. Consistency between the orientations of *r *and *j *depends on whether or not the library preparation protocol preserves the strand information. For single reads *O_a _*= 1 when reads are generated from fragment ends randomly or, for directional RNA-Seq, when they match the known isoform orientation. For paired-end reads, *O_a _*= 1 if the two reads come from different strands, point to each other, and, in the case of directional RNA-Seq, the orientation of first read matches the known isoform orientation.

### The IsoEM algorithm

The IsoEM algorithm starts with the set of *N *known isoforms. For each isoform we denote by *l *(*j*) its length and by *f *(*j*) its (unknown) frequency. If we denote by *n *(*j*) the number of reads coming from isoform *j *and let *p*(*k*) denote the probability of a fragment of length *k*, then(1)

since, the number of fragments of length *k *is expected to be proportional to the number of valid starting positions for a fragment of that length in the isoform. Thus, if the isoform of origin is known for each read, the maximum likelihood estimator for *f*(*j*) is given by *c*(*j*)/(*c*(1) + ... + *c*(*N*)), where  denotes the length-normalized fragment coverage. Note that the length of most isoforms is significantly larger than the mean fragment length *μ *typical of current sequencing libraries; for such isoforms  and *c*(*j*) can be approximated by *n*(*j*)/(*l*(*j*) *μ *+ 1).

**Figure 2:**
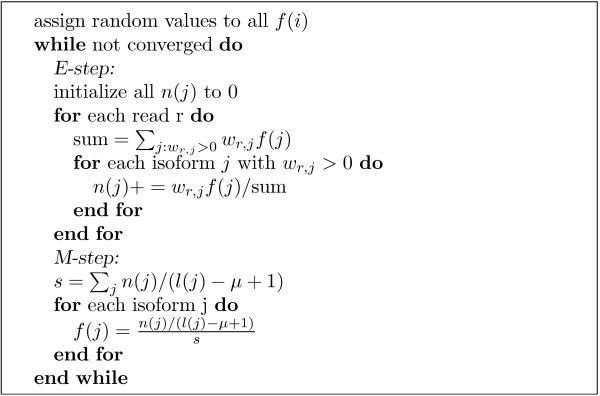
**The expectation-maximization algorithm used by IsoEM**.

Since some reads match multiple isoforms, their isoform of origin cannot be established unambiguously. The IsoEM algorithm (see Figure 2) overcomes this difficulty by simultaneously estimating the frequencies and imputing the missing read origin within an iterative framework. After initializing frequencies *f *(*j*) at random, the algorithm repeatedly performs the next two steps until convergence:

• E-step: Compute the expected number *n*(*j*) of reads that come from isoform *j *under the assumption that isoform frequencies *f*(*j*) are correct, based on weights *w_r, j _*computed as described in the previous section

• M-step: For each *j*, set the new value of *f*(*j*) to *c*(*j*)/(*c*(1) + ... + *c*(*N*)), where normalized coverages *c*(*j*) are based on expected counts computed in the prior E-step

### IsoEM optimizations

Below we describe two implementation optimizations that significantly improve the performance of IsoEM by reducing both runtime and memory usage.

**Figure 3:**
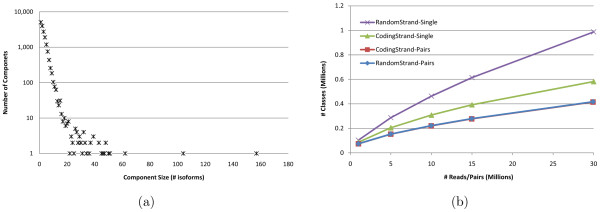
**Distribution of compatibility component sizes (defined as the number of isoforms) for 10 million single reads of length 75 (a) and number of read classes for 1 to 30 million single reads or pairs of reads of length 75 (b)**.

The first optimization consists of partitioning the input into compatibility components. The compatibility between reads and isoforms naturally induces a bipartite read-isoform compatibility graph, with edges connecting each isoform with all reads that can possibly originate from it. Connected components of the compatibility graph can be processed independently in IsoEM since the frequencies of isoforms in one connected component do not affect the frequencies of isoforms in any other connected component. Although this optimization can be applied to any EM algorithm, its impact is particularly significant in IsoEM. Indeed, in this context the compatibility graph decomposes in numerous small components (see Figure 3(a) for a typical distribution of component sizes; a similar distribution of component sizes is reported for Arabidopsis gene models in [27]). The resulting speed-up comes from the fact that in each iteration of IsoEM we update frequencies of isoforms in a single compatibility component, avoiding needless updates for other isoforms.

The second IsoEM optimization consists of partitioning the set of reads within each compatibility component into equivalence classes. Two reads are equivalent for IsoEM if they are compatible with the same set of isoforms and their compatibility weights to the isoforms are proportional. Keeping only a single representative from each read class (with appropriately adjusted frequency) drastically reduces the number of reads kept in memory (see Figure 3(b)). As the number of reads increases, the number of read classes increases much slower. Eventually this reaches saturation and no new read classes appear - at which point the runtime of IsoEM becomes virtually independent of the number of reads. Indeed, in practice the runtime bottlenecks are parsing the reads, computing the compatibility graph and detecting equivalent reads.

**Figure 4:**
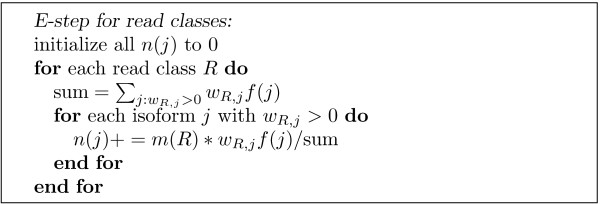
**The E-Step of IsoEM algorithm based on read classes**.

Once read classes are constructed, we only need a small modification of the E-step of IsoEM to use read classes instead of reads (Figure 4). Next we describe the union-find algorithm used for efficiently finding compatibility components and read classes in IsoEM. A read class is defined as 〈*m*,{(*i*, *w*) *i *= isoform, *w *= weight}〉, where *m *is called the multiplicity of the read class. Given a collection of reads, we want to:

• Find the connected components of the compatibility graph induced by the reads, and

• Collapse equivalent reads into read classes with multiplicity indicating the number of reads in each class.

A straightforward approach is to solve the first problem using a union-find algorithm, then to take the reads corresponding to each connected component and remove equivalent reads, e.g., using hashing. However, there are two drawbacks to this approach:

• First, all reads need to be kept in memory until all connected components have been computed.

• Second, when the number of reads in a connected component is very large the number of collisions increases, which leads to poor performance.

We overcome the two problems presented above using an online version of the union-find algorithm which computes connected components and eliminates equivalent reads on the fly. This way, equivalent reads will never reside too long in memory. Also, we avoid the problem of large hash tables by using multiple smaller hash tables which are guaranteed to be disjoint.

We start our modified version of union-find with an empty set of trees. A new single-node tree is initialized every time a new isoform is found in a read class. In each node we store a hash-table of read classes. Each read is processed as follows:

• *If the isoforms compatible with the read correspond to nodes in more than one tree *unite the corresponding trees. The root of the tallest tree becomes the root of the union tree. Then create a new read class for this read (we can be sure it was not seen before, otherwise the isoforms would have been in the same tree) and add it to the hash table of the root node. Notice that at this point the root node is also (trivially) the Lowest Common Ancestor (LCA) of the nodes corresponding to the isoforms in the read class.

• *If the isoforms correspond to nodes in the same tree *find the LCA of all these nodes. If the class of the read is present in the hash table of the LCA, increment its multiplicity and then drop the read. Otherwise, create a new read class and add it to the LCA's hash table.

Notice that in the second case it suffices to look only in the LCA of the isoforms for an already existing read class. This follows immediately from the fact that we always add reads to the LCA of the nodes (isoforms) compatible with the read. Note that we cannot use path compression to speed up 'find' operations because this would be altering the structure of existing trees. Thus, 'find' operations will take logarithmic (amortized) time. At the end of the algorithm, each tree in the union-find forest corresponds to a connected component. The read classes in each connected component are obtained by traversing the corresponding tree and collecting all the read classes present in the nodes. At this point we are sure that all the read classes are distinct, so the collection process performs simple concatenations. To further speed up the collection process, we can safely use path compression as we traverse the trees, since we no longer care about the exact topology of the subtrees.

#### Runtime analysis

Each union operation takes *O*(1) time, so for a read with k compatible isoforms we spend at most *O*(*k*) time doing unions. By always making the root of the taller tree to be the root of a union, we ensure that the height of any tree is not bigger than *O*(log *n*) where *n *is the number of nodes in the tree. Thus, finding the root of a node's tree takes *O*(log *n*). For a read with *k *compatible isoforms we spend at most *O*(*k *log *n*) time processing it. The LCA of two nodes can be computed at constant overhead when performing find operations (by marking the nodes on the paths from isoforms to root). Collecting all the read classes is sped-up by using path compression. The whole collecting phase takes *O*(*nα *(*n*)) time where *n *is the total number of isoforms and *α *(*n*) is the inverse of the Ackermann function. Overall, for *q *reads with an average of *k *isoforms per read and *n *total distinct isoforms, computing read classes and compatibility components using the modified union-find algorithm takes *O*(*qk *log *n *+ *nα *(*n*)) time.

### Hexamer and repeat bias corrections

As noted in [28], some commonly used library preparation protocols result in biased sampling of fragments from isoforms due to the random hexamers used to prime reverse transcription. To correct for possible hexamer bias, we implemented a simple re-weighting scheme similar to that proposed in [28]. Each read is assigned a weight *b*(*h*) based on its first six bases and computed as follows. Given a set of mapped reads, let  be the observed distribution of hexamers starting at position *i *(spanning positions *i *to *i *+ 5) of all the reads. Thus,  is the proportion of reads which have hexamer *h *at position *i *and  is the proportion of reads starting with hexamer *h*. Let *l *be the read length. We define the weights *b *by:

Since we already collapse equivalent reads into read classes, we can seamlessly incorporate hexamer weights in the algorithm by slightly changing the definition of a read class' multiplicity to , where *h*(*r*) denotes the starting hexamer of *r*. The effect of this correction procedure is to reduce (respectively increase) the multiplicity of reads with starting hexamers that are overrepresented (respectively underrepresented) at the beginning of reads compared to the middle of reads. The underlying assumption is that the average frequency with which a hexamer appears in the middle of reads is not affected by library preparation biases. Recent methods [29] also target biases surrounding the start site of the read in addition to within reads.

To avoid biases from incorrectly mapped reads originating from repetitive regions, IsoEM will also discard reads that overlap annotated repeats. When applying this correction, isoform lengths are automatically adjusted by subtracting the number of positions resulting in reads that would be discarded.

## Experimental results

### Comparison of methods on simulated datasets

**Figure 5:**
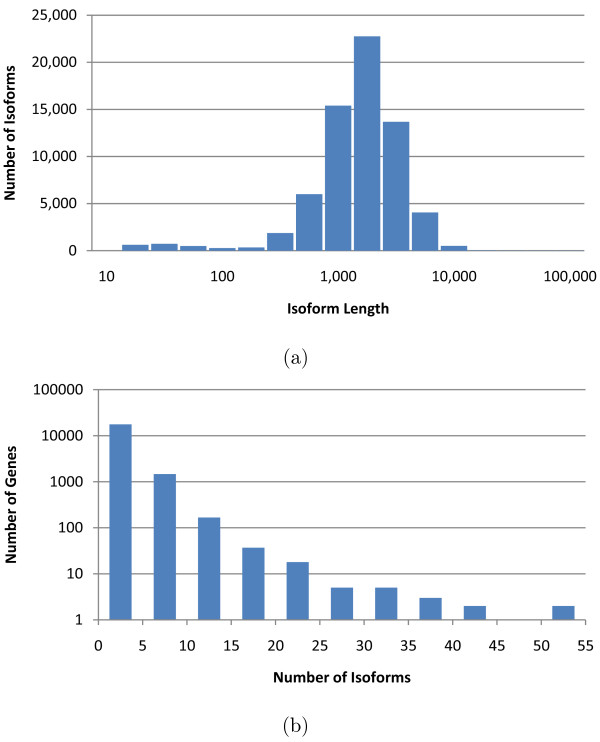
**Distribution of isoform lengths (a) and gene cluster sizes (b) in the UCSC dataset**.

We tested IsoEM on simulated human RNA-Seq data. The human genome sequence (hg18, NCBI build 36) was downloaded from UCSC together with the coordinates of the isoforms in the KnownGenes table. Genes were defined as clusters of known isoforms defined by the GNFAtlas2 table. The dataset contains a total of 66, 803 isoforms pertaining to 19, 372 genes. The isoform length distribution and the number of isoforms per genes are shown in Figure 5.

Single and paired-end reads were randomly generated by sampling fragments from the known isoforms. Each isoform was assigned a *true frequency *based on the abundance reported for the corresponding gene in the first human tissue of the GNFAtlas2 table, and a probability distribution over the isoforms inside a gene cluster. Thus, the true frequency of isoform *j *is *a*(*g*)*p*(*j*), where *a*(*g*) is the abundance of the gene *g *for which *j *is an isoform and *p*(*j*) is the probability of isoform *j *among all the isoforms of *g*. We simulated datasets with uniform, respectively truncated geometric distribution with ratio *r *= 1/2 for the isoforms of each gene. For a gene with *k *isoforms *p*(*j*) = 1/*k*, *j *= 1, ..., *k*, under the uniform distribution. Under the truncated geometric distribution, the respective isoform probabilities are *p*(*j*) = 1/2^*j *^for *j *= 1, ..., *k *- 1 and *p*(*k*) = 1/2^*k*-1^. Fragment lengths were simulated from a normal probability distribution with mean 250 and standard deviation 25.

We compared IsoEM to several existing algorithms for solving the IE and GE problems. For IE we included in the comparison the isoform analogs of the Uniq and Rescue methods used for GE [9], an improved version of Uniq (UniqLN) that estimates isoform frequencies from unique read counts but normalizes them using adjusted isoform lengths that exclude ambiguous positions, the Cufflinks algorithm of [20] (version 0.8.2), and the RSEM algorithm of [13] (version 0.6). For the GE problem, the comparison included the Uniq and Rescue methods, our implementation of the GeneEM algorithm described in [10], and estimates obtained by summing isoform expression levels inferred by Cufflinks, RSEM, and IsoEM. All methods use alignments obtained by mapping reads onto the library of isoforms with Bowtie [26] and then converting them to genome coordinates, except for Cufflinks which uses alignments obtained by directly mapping the reads onto the genome with TopHat [21], as suggested in [20].

Frequency estimation accuracy was assessed using the coefficient of determination, *r*^2^, along with the *error fraction (EF) and median percent error (MPE) *measures used in [13]. However, accuracy was computed against true frequencies, not against estimates derived from true counts as in [13]. If  is the frequency estimate for an isoform with true frequency *f_i_*, the *relative error *is defined as  if *f_i _*≠ 0, 0 if , and ∞ if . The error fraction with threshold *τ*, denoted *EF_τ _*is defined as the percentage of isoforms with relative error greater or equal to *τ*. The median percent error, denoted MPE, is defined as the threshold *τ *for which *EF_τ _*= 50%.

Since not all compared methods could handle paired reads or strand information we focused our comparisons on single read data. Table 1 gives *r*^2 ^values for isoform, respectively gene expression levels inferred from 30 M reads of length 25, simulated assuming both uniform and geometric isoform expression. IsoEM significantly outperforms the other methods, achieving an *r*^2 ^values of over .96 for all datasets. For all methods the accuracy difference between datasets generated assuming uniform and geometric distribution of isoform expression levels is small, with the latter one typically having a slightly worse accuracy. Thus, in the interest of space we present remaining results only for datasets generated using geometric isoform expression.

**Table 1:** *r*^2 ^for isoform and gene expression levels inferred from 30 M reads of length 25 from reads simulated assuming uniform, respectively geometric expression of gene isoforms.

Isoform Expression			Gene Expression		
**Algorithm**	**Uniform**	**Geometric**	**Algorithm**	**Uniform**	**Geometric**

Uniq	0.466	0.447	Uniq	0.579	0.586
Rescue	0.693	0.675	Rescue	0.724	0.724
UniqLN	0.856	0.838	GeneEM	0.636	0.637
Cufflinks	0.661	0.618	Cufflinks	0.778	0.757
RSEM	0.919	0.911	RSEM	0.939	0.934
IsoEM	**0.971**	**0.970**	IsoEM	**0.990**	**0.982**

**Figure 6:**
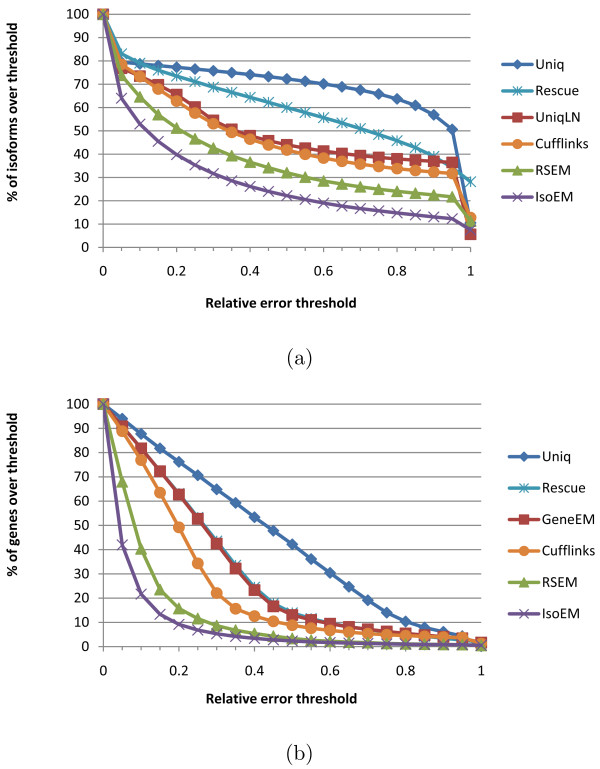
**Error fraction at different thresholds for isoform (a) and gene (b) expression levels inferred from 30 M reads of length 25 simulated assuming geometric isoform expression**.

For a more detailed view of the relative performance of compared IE and GE algorithms, Figure 6 gives the error fraction at different thresholds ranging between 0 and 1. The variety of methods included in the comparison allows us to tease out the contribution of various algorithmic ideas to overall estimation accuracy. The importance of rigorous length normalization is illustrated by the significant IE accuracy gain of UniqLN over Uniq - clearly larger than that achieved by ambiguous read reallocation as implemented in the IE version of Rescue. Proper length normalization is also explaining the accuracy gain of isoform-aware GE methods (Cufflinks, RSEM, and IsoEM) over isoform oblivious GE methods. Similarly, the importance of modeling insert sizes even for single read data is underscored by the significant IE and GE accuracy gains of IsoEM over RSEM. Indeed, the latest version of the RSEM package, released as this article goes to print, has been updated to include modeling of insert sizes and appears to have accuracy matching that of IsoEM.

For yet another view, Tables 2 and 3 report the MPE and EF_.15 _measures for isoform, respectively gene expression levels inferred from 30 M reads of length 25, computed over groups of isoforms with various expression levels. IsoEM consistently outperforms the other IE and GE methods at all expression levels except for isoforms with zero true frequency, where it is dominated by the more conservative Uniq algorithm and its UniqLN variant.

**Table 2:** Median percent error (MPE) and 15% error fraction (EF_.15_) for isoform expression levels inferred from 30 M reads of length 25 simulated assuming geometric isoform expression.

Expression range		0	(0, 10^-6^]	(10^-6^, 10^-5^]	(10^-5^, 10^-4^]	(10^-4^, 10^-3^]	(10^-3^, 10^-2^]	All
# isoforms		13,290	10,024	23,882	18,359	1,182	66	66,803
	Uniq	**0.0**	**100.0**	98.4	97.1	98.5	96.6	95.4
	Rescue	**0.0**	294.7	75.5	49.2	30.4	28.3	71.9
MPE	UniqLN	**0.0**	**100.0**	80.8	30.3	26.4	24.8	36.0
	Cufflinks	**0.0**	**100.0**	49.7	25.5	27.2	44.6	34.1
	RSEM	**0.0**	**100.0**	31.9	13.5	11.4	13.0	21.2
	IsoEM	**0.0**	**100.0**	**25.3**	**7.3**	**3.2**	**2.2**	**12.0**

	Uniq	**0.2**	98.4	97.2	96.9	97.0	95.5	78.0
	Rescue	48.4	95.5	86.2	73.1	61.5	56.1	76.0
^EF^.15	UniqLN	**0.2**	97.2	86.2	82.8	83.3	77.3	69.8
	Cufflinks	17.6	96.4	81.3	71.0	74.7	80.3	67.9
	RSEM	19.9	93.7	71.1	46.4	39.8	47.0	56.9
	IsoEM	3.4	**93.1**	**65.1**	**29.1**	**11.1**	**7.6**	**46.1**

**Table 3:** Median percent error (MPE) and 15% error fraction (EF_.15_) for gene expression levels inferred from 30 M reads of length 25 simulated assuming geometric isoform expression.

Expression range		(0, 10^-6^]	(10^-6^, 10^-5^]	(10^-5^, 10^-4^]	(10^-4^, 10^-3^]	(10^-3^, 10^-2^]	All
# genes		120	5,610	11,907	1,632	102	19,372
	Uniq	37.4	43.6	42.7	43.0	48.2	43.0
	Rescue	32.8	28.7	26.0	25.1	28.8	26.7
MPE	GeneEM	30.6	28.2	25.7	25.1	28.0	26.3
	Cufflinks	33.0	21.1	19.0	20.2	40.2	19.7
	RSEM	23.6	11.0	7.2	7.9	11.4	8.1
	IsoEM	**18.2**	**8.4**	**3.2**	**2.0**	**1.9**	**3.9**

	Uniq	77.5	82.4	81.7	79.7	82.4	81.7
	Rescue	74.2	74.0	71.6	72.8	76.5	72.4
EF.15	GeneEM	72.5	73.8	71.5	73.0	74.5	72.3
	Cufflinks	73.3	64.7	62.3	66.2	82.3	63.5
	RSEM	64.2	37.3	17.4	16.3	41.2	23.5
	IsoEM	**57.5**	**28.1**	**6.7**	**6.1**	**4.9**	**13.2**

### Comparison of methods on two real RNA-Seq datasets

In addition to simulation experiments, we validated IsoEM on two real RNA-Seq datasets. The first dataset consists of two samples with approximately 8 million 27 bp Illumina reads each, generated from two human cell lines (embryonic kidney and B cells) as described in [30]. Estimation accuracy was assessed by comparison with quantitative PCR (qPCR) expression levels determined in [14] for 47 genes with evidence of alternative isoform expression. To facilitate comparison with these qPCR results, expression levels were determined using transcript annotations in ENSEMBL version 46. The second dataset consists of approximately 5 million 32 bp Illumina reads per sample, generated from the RM11-1a strain of *S*. *cerevisiae *under two different nutrient conditions [31]. Expression levels were determined using transcript annotations for the reference strain (June 2008 SGD/sacCer2) and compared against qPCR expression levels measured for 192 genes (for a total of 394 datapoints).

**Figure 7:**
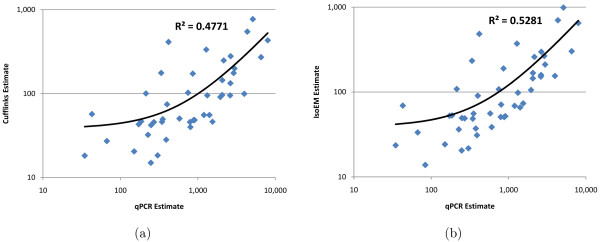
**Comparison of Cufflinks (a) and IsoEM (b) estimates to qPCR expression levels reported in **[14].

**Figure 8:**
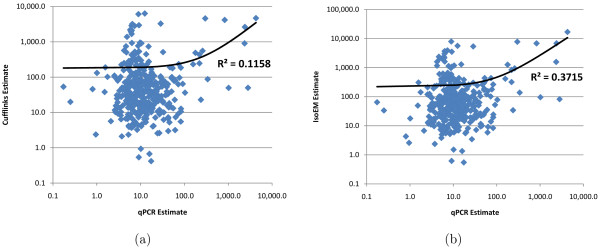
**Comparison of Cufflinks (a) and IsoEM (b) estimates to qPCR expression levels reported in **[31].

Since the available implementation of RSEM could not be run on transcript sets other than UCSC known genes, in Figures 7 and 8 we only compare Cufflinks and IsoEM estimates against qPCR values in [14], respectively [31]. Estimation accuracy of both Cufflinks and IsoEM is significantly lower than that observed in simulations. Likely explanations include poor quality of the transcript libraries used to perform the inference, sequencing library preparation biases not corrected for by the algorithms, and possible inaccuracies in qPCR estimates. Nevertheless, the relative performance of the two algorithms is consistent with simulation results, with IsoEM outperforming Cufflinks on both datasets.

### Influence of sequencing parameters and scalability

**Figure 9:**
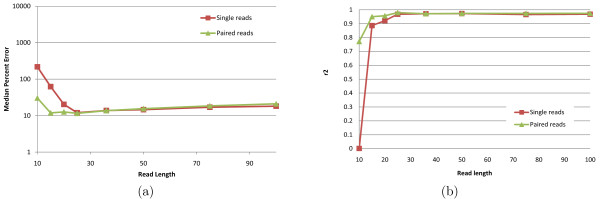
**IsoEM MPE (a) and *r*^2 ^values (b) for 750 Mb of simulated data generated using single and paired-end reads of length varying between 10 and 100**.

**Figure 10:**
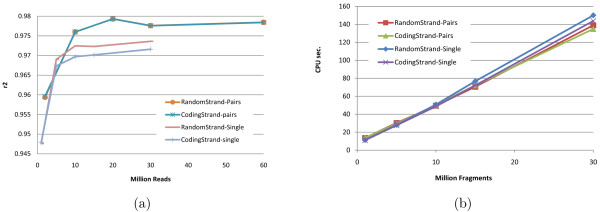
**IsoEM *r*^2 ^(a) and CPU time (b) for 1-60 million single/paired reads of length 75, with or without strand information**.

Although high-throughput technologies allow users to make tradeoffs between read length and the number of generated reads, very little has been done to determine optimal parameters even for common applications such as RNA-Seq. The intuition that longer reads are better certainly holds true for many applications such as *de novo *genome and transcriptome assembly. Surprisingly, [13] found that *shorter *reads are better for IE when the total number of sequenced bases (as a rough approximation for sequencing cost) is fixed. Figure 9 plots IE estimation accuracy for reads of length between 10 and 100 when the total amount of sequence data is kept constant at 750 M bases. Our results confirm the finding of [13], although the optimal read length is somewhat sensitive to the accuracy measure used and to the availability of pairing information. While 25 bp reads minimize MPE regardless of the availability of paired reads, the read length that maximizes *r*^2 ^is 25 for paired reads and 50 for single reads. Although further experiments are needed to determine how the optimum length depends on the amount of sequence data and transcriptome complexity, our simulations do suggest that for isoform and gene expression analysis, increasing the number of reads may be more useful than increasing read length beyond 50 bases. Figure 10(a) shows, for reads of length 75, the effects of paired reads and strand information on estimation accuracy as measured by *r*^2^. Not surprisingly, for a fixed number of reads, paired reads yield better accuracy than single reads. Also not very surprisingly, adding strand information to paired sequencing yields no benefits to genome-wide IE accuracy (although it may be helpful, e.g., in identification of novel transcripts). Quite surprisingly, performing strand-specific single read sequencing is actually *detrimental *to IsoEM IE (and hence GE) accuracy under the simulated scenario, most likely due to the reduction in sampled transcript length.

In practice, many RNA-Seq data sets are generated from transcripts with poly(A) tails, and some of the sequenced fragments will contain parts of the poly(A) tails. We have added to IsoEM the option to automatically extend annotated transcripts with a poly(A) tail, thus allowing it to use reads coming from such fragments. Table 4 shows the accuracy of isoform and gene expression levels inferred by IsoEM using 30 M reads of length 25 simulated from transcripts with and without poly(A) tails assuming geometric expression of gene isoforms. The accuracy of IsoEM is practically the same under the two simulation scenarios for paired read data, and decreases only slightly for single reads simulated taking poly(A) tails into account, likely due to the fact that reads overlapping poly(A) tails are more ambiguous.

**Table 4:** *r*^2 ^for isoform and gene expression levels inferred from 30 M single, respectively paired reads of length 25, simulated assuming geometric expression of gene isoforms with and without poly(A) tails.

Reads	Poly(A)	Isoform Expression	Gene Expression
1 × 25	Yes	0.956	0.977
	No	0.970	0.982

2 × 25	Yes	0.972	0.990
	No	0.976	0.985

As shown in Figure 10(b), the runtime of IsoEM scales roughly linearly with the number of *fragments*, and is practically insensitive to the type of sequencing data (single or paired reads, directional or non-directional). IsoEM was tested on a Dell PowerEdge R900 server with 4 Six Core E7450Xeon Processors at 2.4 Ghz (64 bits) and 128 Gb of internal memory. None of the datasets required more than 16 GB of memory to complete. It is also true that increasing the available memory significantly decreases runtime by keeping the garbage collection overhead to a minimum. The runtimes in Figure 10 were obtained by allowing IsoEM to use up to 32 GB of memory, in which case none of the datasets took more than 3 minutes to solve.

## Conclusions and ongoing work

In this paper we have introduced an expectation-maximization algorithm for isoform frequency estimation assuming a known set of isoforms. Our algorithm, called IsoEM, explicitly models insert size distribution, base quality scores, strand and read pairing information. Experiments on both real and synthetic RNA-Seq datasets generated using two different assumptions on the isoform distribution show that IsoEM consistently outperforms existing algorithms for isoform and gene expression level estimation with respect to a variety of quality metrics.

The open source Java implementation of IsoEM is freely available for download at http://dna.engr.uconn.edu/software/IsoEM/. In ongoing work we are extending IsoEM to perform allelic specific isoform expression and exploring integration of isoform frequency estimation with identification of novel transcripts using the iterative refinement framework proposed in [18].

## Competing interests

The authors declare that they have no competing interests.

## Authors' contributions

IIM and AZ conceived the study. MN designed and implemented the algorithms and drafted the manuscript along with IIM. MN and SM conducted the experiments. All authors participated in data analysis and manuscript revision. All authors have read and approved the final manuscript.
